# Understanding Intratumor Heterogeneity and Evolution in NSCLC and Potential New Therapeutic Approach

**DOI:** 10.3390/cancers10070212

**Published:** 2018-06-22

**Authors:** Taichiro Goto, Yosuke Hirotsu, Kenji Amemiya, Hitoshi Mochizuki, Masao Omata

**Affiliations:** 1 Lung Cancer and Respiratory Disease Center, Yamanashi Central Hospital, Kofu 400-8506, Japan; 2 Genome Analysis Center, Yamanashi Central Hospital, Kofu 400-8506, Japan; hirotsu-bdyu@ych.pref.yamanashi.jp (Y.H.); amemiya-bdcd@ych.pref.yamanashi.jp (K.A.); h-mochiduki2a@ych.pref.yamanashi.jp (H.M.); m-omata0901@ych.pref.yamanashi.jp (M.O.)

**Keywords:** intratumor heterogeneity, cancer genome evolution, targeted therapy

## Abstract

Advances in innovative technology, including next-generation sequencing, have allowed comprehensive genomic analysis and the elucidation of the genomic aspect of intratumor heterogeneity (ITH). Moreover, models of the evolution of the cancer genome have been proposed by integrating these analyses. Cancer has been considered to accumulate genetic abnormalities for clonal evolution in time and space, and these evolutionary patterns vary depending on the organs of primary sites. Selection pressure is an important determinant of such evolutionary patterns. With weak selection pressure, more diverse clones coexist, and heterogeneity increases. Heterogeneity is maximized when there is no selection pressure; in other words, neutral evolution occurs. Some types of cancer such as lung cancer evolve in conditions that have maintained close to neutral evolution and produce diverse variants. This ITH is a key factor contributing to the lethal outcome of cancer, therapeutic failure, and drug resistance. This factor reaffirms the complexity and subtle adaptability of cancer. It is expected that further understanding of ITH and cancer genome evolution will facilitate the development of new therapeutic strategies to overcome ITH.

## 1. Introduction

Heterogeneity or diversity in groups of individuals is a phenomenon that is observed in the natural world. Genomic diversity and interindividual variation are potent survival strategies to adapt to environmental changes. It has been elucidated that cancer cells also use these strategies artfully.

Genomic diversity within single tumors has been recognized for a long time; however, it is only with the advent of next-generation sequencing studies that the complete extent of genomic intratumor heterogeneity (ITH) is becoming apparent [1,2,3,4,5,6,7,8]. The sequencing of spatially or temporally distinct tumor regions has begun to uncover the bewildering extent of diversity within tumors [1,3].

The process of temporal and spatial changes in the cancer genome is one of the important molecular mechanisms underlying heterogeneity in cancer [1,3]. Many types of cancer are known to be caused by the accumulation of multiple genomic abnormalities [9]. The resulting presence of clonal tumor cells implies that a population of cells with specific genomic abnormalities proliferates predominantly [9]. Since this phenomenon resembles adaptive evolution, which is observed in the natural world, it has been termed cancer genome evolution [10]. Adaptive evolution originally refers to a process to develop features that are beneficial for the survival and reproduction of individuals [11]. However, in cancer, adaptive evolution refers to an improvement in the proliferation speed and adaptation to environmental changes such as hypoxic conditions, low nutrition, and chemotherapy [10,12].

The advances in next generation sequencing have made it possible to powerfully analyze tumor evolution, which in turn has improved our understanding of the initiation and development of tumors, as well as the interaction between cancer cells and the immune microenvironment. In this review, we explore the extent and clinical implications of ITH, and discuss the profiling of the genetic diversity of tumors to trace their life histories and patterns of evolution. It is important to view tumor development in the context of a dynamic evolutionary framework as well as the interaction of tumor cells with the microenvironment. Finally, we propose new approaches to treat cancer systemically by considering ITH and tumor evolution.

## 2. Intratumor Heterogeneity and Pattern of Tumor Development

**Figure 1:**
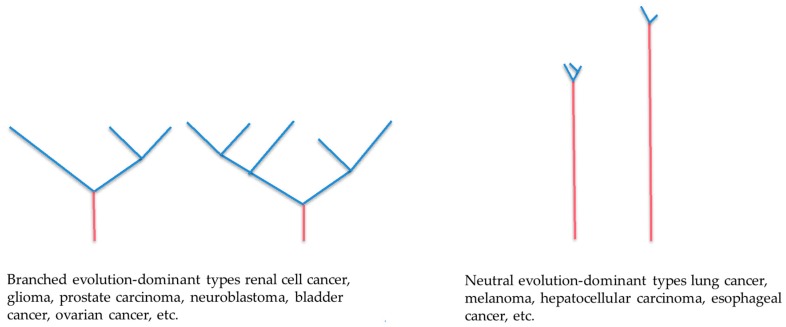
Phylogenetic trees for the cancer genome. These schemas of genomic evolutionary trees indicate how cancer evolves through branched evolution and neutral evolution. The red and blue line represents a common mutation and a subclone mutation, respectively. The branch length indicates evolutionary distance and is correlated with the number of nucleotide substitutions.

Based on the genomic analyses performed for multiple regions of a tumor, phylogenetic trees can be constructed to determine the cancer genome evolution in each case to reveal how the cancer has evolved [1,3,13,14]. McGranahan et al. have reported typical phylogenetic trees for each type of carcinoma (Figure 1) [15]. According to these trees, carcinomas can be divided into two groups: a group characterized by wide gaps between branches, and a group of carcinomas sharing common genomic abnormalities and characterized by a short distance from the branching point [15]. The former often follows branched evolution, whereas the latter often follows neutral evolution.

### 2.1. Branched Evolution

When driver genes simultaneously mutate in two different clones and remain effective, the clones are sometimes so comparable that neither drives out the other. Instead, they may evolve further independently. This evolutionary pattern is referred to as branched evolution (Figure 1). In this pattern, the mutations of key important driver genes for tumorigenesis show local heterogeneity. Gerlinger et al. sequenced and analyzed genes in multiple regions of renal cancer, and demonstrated that mutations in several tumor suppressor genes located in chromosome 3 (SEDT2, PTEN, and KDM5C) differ among regions within the same tumor, indicating branched evolution [16]. Such cases have also been reported for several other types of cancer, such as mutations in the epidermal growth factor receptor gene in glioblastoma and NOTCH1 in esophageal cancer [17,18].

This branched evolution is often observed even after molecular-targeted therapy. For example, in a study on BRAF-mutated malignant melanoma, treatment of BRAF-inhibitor led to the proliferation of clones of KRAS mutation, BRAF gene amplification, and PTEN gene loss, at different sites within a lesion [19].

### 2.2. Neutral Evolution

**Figure 2:**
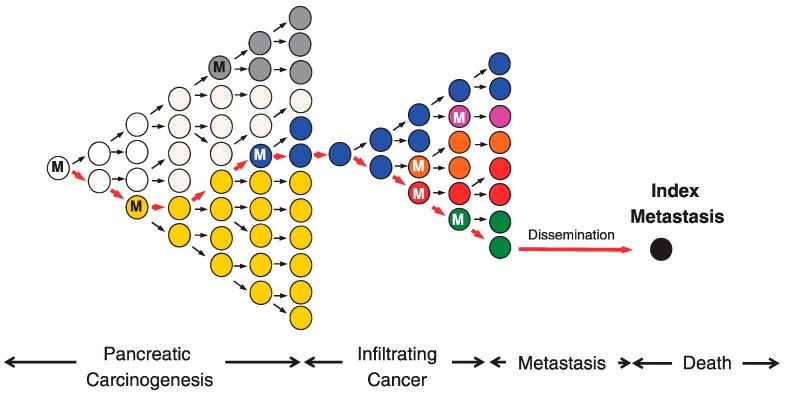
Clonal evolution of pancreatic carcinogenesis and progression. Red arrows indicate the lineage of the index metastasis from its origin in a normal cell. Carcinogenesis begins with an initiating alteration (M) in a normal cell that provides a selective advantage. Before the establishment of fixed cancer cells that are referred to as parental clones, clones with mutations that are beneficial for adapting to the environment are selected by selection pressure and survive. This clonal expansion is expected to generate more than one subclone within a cancer, one of which will give rise to the founder cell (blue clone) that will eventually become the parental clone and hence initiate the infiltrating carcinoma. Once parental clones are established, various spontaneous mutations seem to be accumulated. Even mutations that are not necessarily beneficial in the microenvironment are accumulated. This state refers to intratumor heterogeneity (ITH), which is observed clinically. While abnormal genes are accumulated, subclones that can adapt to the microenvironment at metastatic sites appear and metastasize (black clone). M: Gene mutation. This figure is adapted from Yachida S. et al. [22].

In evolution in the biosphere, an increase in the proportion of some individual or phenotype in a population is referred to as being selected, and the factor causing the difference is referred to as selection pressure [11]. Sottoriva et al. analyzed several regions of colorectal cancer and found that, in advanced cancer, clones with acquired mutations coexist and proliferate without being apparently selected and eliminated [20]. They thus proposed a so-called big bang of diversity model (Figure 1). Furthermore, using the cancer genome data from 904 cases, including 14 types of cancer, Sottoriva et al. revealed that the mutations detected in approximately 30% of the cases were neutral (not affected by strong selection pressure) [21]. In an early carcinogenic stage of neutral evolution, precancerous cells are repeatedly selected and eliminated until tumor cells are established [22]. Once established, additional mutations occurring in the clones of established tumor cells in later stages are neutral (Figure 2) [22].

**Figure 3:**
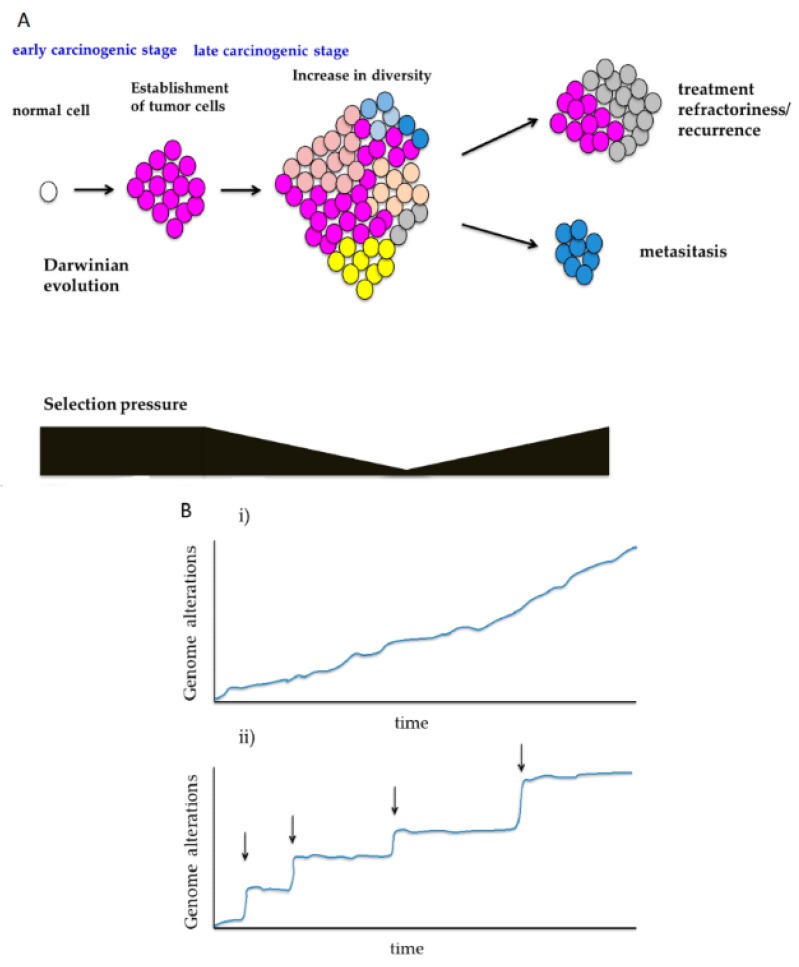
Temporal changes in diversity during cancer genome evolution. (**A**) Changes in selection pressure during carcinogenesis: Before the establishment of parental clones containing the minimum required abnormal driver genes, cancer cells are under a certain level of selection pressure. Afterwards, selection pressure is weakened, and genetic diversity in the lesions increases. In refractory or metastatic lesions, many different selection pressures are generated. (**B**) Diversity in adaptive evolution: (i) Sequential evolution: the exemplary pattern of adaptive evolution in which phenotypes are gradually obtained by spontaneous mutations and other processes. (ii) Punctuated evolution: the exemplary pattern of adaptive evolution characterized by discontinuous and distinctive acquisition of phenotypes (arrows).

In the evolutionary theory, neutral evolution refers to the absence of any population evolving through apparent adaptive evolution [11]. In cancer, when clones with specific mutations are affected by strong positive or negative selection pressure, the frequency of mutations deviates from that in the neutral evolution pattern. If no significant deviation is observed, tumor cells may have neutrally evolved without being affected by selection pressure. In the field of molecular evolution, a commonly used index for assessing selection pressure is the Ka/Ks ratio (Ka: DNA substitution rate of non-synonymous mutation; Ks: DNA substitution rate of synonymous mutation) [23,24,25,26]. Based on calculations using the cancer genomic data from The Cancer Genome Atlas (TCGA), it has been reported that there are extremely few driver genes with a Ka/Ks ratio of more than one (affected by positive selection pressure) [27]. In cases where positive and negative selection pressures are almost comparable, the Ka/Ks ratio can be one, though such a condition rarely exists. It is assumed that, in many cancer cases, the mutations generated at every cell division mostly coexist neutrally as passenger mutations. Such neutral mutations increase cell populations with more diverse genomic mutations, and are an important driving force for the heterogeneity in cancer (Figure 3A) [1,3,21,28].

It is evident that certain tumor types, such as melanoma and lung cancer, harbor a significantly larger mutational burden than other types, and they can be regarded as a typical example of neutral evolution among many types of carcinomas [15,21]. The involvement of powerful exogenous mutagens, such as ultraviolet light and tobacco carcinogens, which a stem cell niche may be exposed to for years during the early carcinogenic stage, likely explains the presence of strong and solitary driver mutation and the high mutation burden in these cancers [15].

Since lung cancer is a neutrally evolving tumor, it has been clinically demonstrated that the driver mutation can be a clonal marker of lung cancer. In cases of multiple lung cancers, the comparison of driver mutation profiles clarifies the clonal origin of the tumors and enables discrimination between the primary and metastatic tumors [29,30].

## 3. Temporal Diversity in the Cancer Genome Evolutionary Processes

The various cancer genome evolutionary processes described above also change over time, which contributes to the heterogeneity in cancer.

### 3.1. Shifts in Selection Pressure During Carcinogenesis

The temporal and spatial diversity in selection pressure greatly affects the evolutionary process. Thus, it seems common that in the early stages of carcinogenesis, branched evolution is observed because specific driver genes are selected under stronger selection pressure, and that in the late stages of carcinogenesis, neutral evolution occurs under weak selection pressure (Figure 2 and Figure 3A) [22].

In 1867, Alfred Russel Wallace summarized Darwinism as, “The fittest will survive, and a race will be eventually produced, adapted to the conditions in which it lives” [11]. Although originally stated in such a way as to apply to the evolution of individual organisms within a population, the principle idea of Darwinian evolution, i.e., variation with differential fitness that is heritable, can be applied to tumor evolution [10]. In the early stages of carcinogenesis, the population of cancer cells is subject to selection (Figure 2) [22]. Conceivably, during Darwinian evolution, both selection and neutral growth may operate simultaneously within the same tumor, and this may alter dynamically over time. The genetic variation between these cells, which is influenced by endogenous and exogenous mutational processes, may lead to the selection of aggressive subclones, one of which will give rise to the founder cell that will eventually become the parental clone and initiate the infiltrating carcinoma (Figure 2) [31]. After the birth of this parental clone, selection pressure decreases, and ITH development can follow the laws of neutral growth (Figure 2 and Figure 3A) [20].

Halazonetis et al. proposed an oncogene-induced DNA damage model for cancer development [32]. According to the model, activated oncogenes induce the formation of DNA double-strand breaks in both precancerous lesions and cancers, which in turn lead to genomic instability and incremental acquisition of genetic mutations in the late carcinogenic stage [32,33].

Meanwhile, because environmental changes such as metastasis and treatment generate new selection pressures, adaptive evolution may additionally occur, even in the late stages of carcinogenesis (Figure 2 and Figure 3A) [34]. Sun et al. identified indices distinguishing between neutral and adaptive evolution through simulation with models of cancer genome evolution, and assessed these indices by applying them to actual cancer genome data [35]. As a result, they reported that evolutionary processes might vary not only among cases, but will vary among regions in the same case, and that adaptive selection is commonly observed in early lesions and posttreatment samples [35].

### 3.2. Sequential Evolution and Punctuated Evolution

Genomic evolution characterized by point mutations follows the following sequence: new mutations are generated at every cell division, and although clone selection may or may not occur (neutral evolution) among new mutations, heterogeneity increases gradually (Figure 3Bi) [1,3].

In contrast, compared with point mutations, abnormalities in chromosome structures such as changes in the number of copies affect many genes, and consequently have a greater impact on clones acquiring such abnormalities [17,28,36,37]. Cancer genome evolution associated with such abnormalities of chromosome structure sometimes follows a discontinuous evolutionary process, which is referred to as punctuated evolution (Figure 3Bii), although punctuated and sequential evolution is certainly not exclusive, and both processes may be observed in a single case. Chromothripsis, which is characterized as a single catastrophic event resulting in tens of hundreds of locally clustered rearrangements affecting one or a few chromosomes, has also been documented to be widespread in some types of cancer, including lung adenocarcinomas, bladder cancers, pancreatic cancers, glioblastomas, and esophageal adenocarcinomas [37,38,39,40]. Chromothripsis is a phenomenon in which some chromosomes are fragmented and reconstructed in a single stage [41]. Such large-scale genomic reconstruction in a short period of time, as well as the appearance of fusion genes that cannot be generated by point mutation, is assumed to generate clones with completely different features before and after the occurrence of abnormalities, and these clones seem to form new evolutionary lineages (Figure 3Bii). These large-scale genomic rearrangements are usually related to the aggressive character of cancer [28]; however, cancers with an extreme level of chromosomal instability tend to exhibit more favorable prognosis, as compared with intermediate levels [42,43,44]. In addition, oncogenic fusion genes caused by chromothripsis may function as driver mutations and thus represent promising therapeutic targets [40,45,46,47].

## 4. Intercellular Interaction in Cancer Genome Evolution

The discussion in the previous sections is based on an assumption that all of the populations of different clones compete with one another, and that clones that successfully adapt to the environment are selected. However, in the actual living world, Darwinian evolution is not necessarily an optimal solution for survival.

Recently, there have also been reports of cancer cases in which several clonal populations are not in a competitive relationship, but rather evolve together while maintaining a cooperative relationship. For the establishment of such networks in some types of ecosystems, it is considered that intercellular interaction between humoral factors (e.g., cytokines) and transmembrane signaling molecules (e.g., ephrin) plays an important role. Cleary et al. reported cooperative and dependent relationships between clones with and without the expression of Wnt1 in a murine breast cancer model [48]. In an experiment with transplant model mice, Marusyk et al. demonstrated that there were subclones contributing to the overall tumor growth through interleukin 11, and that the disappearance of these subclones resulted in a decrease in the overall size of the tumors [49]. Their results suggest that non-cell autonomous drivers may be required for tumor development. In some cases, such a cooperative relationship may be attributable to the hierarchy among clones, as with cancer stem cells. However, ITH, in which several clones cooperate and coexist as seen in actual cases, may also be one of optimal solutions obtained through cancer genome evolution. When the cancer cell society and the host microenvironment are regarded as a single ecosystem, the selection and elimination in cancer genome evolution may be established on a complex balance.

Meanwhile, the immune system, which has recently been attracting much attention in terms of treatment, is also a great influential factor for cancer genome evolution and heterogeneity. As studies on immune checkpoint inhibitors have revealed, the host immune system targets neoantigens generated by cancer genomic mutations [50,51]; therefore, the immune system exerts strong selection pressure on cancer genome evolution. Presumably, mutations exhibiting potent antigenicity are subjected to negative selection pressure to be eliminated by the immune system, whereas mutations associated with immune evasion, such as activation of the PD-L1 (programmed death ligand 1) gene and human leukocyte antigen gene mutations, are subjected to positive selection pressure [21,42,52,53,54,55].

## 5. Degree of Heterogeneity and Limiting Points

For tumors following various genomic evolutionary processes, the factors determining the degree of heterogeneity include DNA instability and the number of cell populations, in addition to selection pressure. In case of the high accumulation of mutations resulting from mismatch repair disturbance, many cells with neutral mutations are generated, so that the degree of heterogeneity increases in the populations [56,57,58,59,60,61,62,63,64,65,66]. On the other hand, as the number of tumor cells increases, the degree of heterogeneity increases equally in the whole tumor. Moreover, the presence of selection pressure induces specific clone populations to proliferate, and consequently reduces the degree of heterogeneity. Thus, because the degree of heterogeneity is higher in larger tumors with deficient mismatch repair but that are almost free from selection pressure, they are likely to adapt to environmental changes such as treatment (acquisition of treatment refractoriness and recurrence).

Is this genomic heterogeneity limitless, or is there a certain limiting point? As mutations accumulate, the probability of occurrence of adverse mutations for cells increases, and cancer cells with excessive accumulation of mutations are more likely to be eliminated as a consequence of harmful gene mutations. Thus, there is presumably a certain upper limit for the number of mutations. In fact, it has been reported that, in POLE (polymerase ε)-mutated cancer, which exhibits a high accumulation of mutations, the upper limit for the total number of spontaneous mutations in gene regions is approximately 20,000 mutations [67]. Excessive abnormalities in chromosomal structure may also be disadvantageous to cells [42,43,44]. A large-scale analysis of TCGA data revealed that the prognosis was rather favorable in patients with copy-number alterations affecting more than 75% of the entire genome [42]. Moreover, in evolutionary therapy, Muller’s ratchet hypothesis indicates that asexually reproducing organisms gradually accumulate harmful genes and become extinct when they become unable to adapt to the environment [68]. Another factor for the presence of an upper limit may be a higher possibility that cancer cells with more genomic mutations are recognized and eliminated by the host immune system as described above. The limiting point of genomic heterogeneity associated with genomic evolution may also need to be assessed in terms of treatment and prognosis in the future.

## 6. Significance of ITH in Treatment Refractoriness, Recurrence, and Metastasis

ITH, which is achieved through the various processes described above, is beneficial for the survival of tumors. Tumors can survive if a heterogeneous cell population contains clones that can resist selection pressures associated with limitations on blood flow, oxygen supply, and space, as well as immunity and treatment. Analyses of specimens obtained from multiple regions during autopsies of patients with bladder or ovarian cancer revealed that recurrence lesions after anticancer or hormonal therapy were derived from clones arising in the very early stages of tumorigenesis [69,70]. These cases suggested that recurrence might be attributed to clones that had escaped the selection pressure imposed by treatment [69,70]. Moreover, studies using murine breast cancer models demonstrated that, when tumors metastasize from primary lesions, metastatic lesions are polyclonal, regardless of any of the following metastatic processes: infiltration, local dissemination, endovascular embolization, circulating tumor cell clusters, and micrometastasis [71]. Thus, the presence of ITH is presumably beneficial for metastasis as well.

### 6.1. Combination of Molecular Targeted Therapy and Immunotherapy

**Figure 4:**
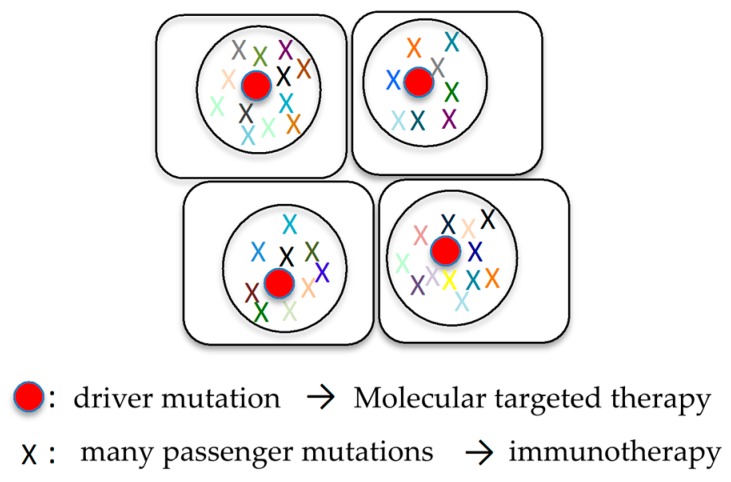
Combination of molecular targeted therapy and immunotherapy. Cancer cells harbor many mutations, typically single driver mutation and many passenger mutations in lung cancer. Driver and passenger mutations can become the therapeutic target for molecular targeted therapy and immunotherapy, respectively.

The establishment of personalized medicine is a goal of cancer treatment. In cancer patients, the cancer genome is analyzed to identify abnormal genes specific to their cancers. To administer personalized treatment to cancer patients, it is doubtful whether the overall molecular genetic features of cancer can be assessed using biopsy specimens obtained from only one site, and the analysis of circulating tumor DNA in plasma is regarded as a promising procedure to study ITH [72,73,74,75]. Screening for recurrence is also performed using circulating tumor DNA, and molecular targeted therapy is frequently administered for abnormal genes that can be therapeutic targets [76,77,78,79,80]. ITH is of great importance when treatment based on such abnormal cancer genes is administered. If passenger mutations are targeted, different subclones without them will survive and cause recurrence or metastasis. Thus, the driver mutations that exist in all cancer cells should be considered as therapeutic targets (Figure 4).

Meanwhile, abnormal proteins produced by mutated genes are presented as neoantigens [50,51]. Immune cells should be activated by them and function to eliminate cancer from the body [50,51]. However, because immune evasion mechanisms are activated through pathways mediated by immune checkpoint-associated molecules such as PD-1, PD-L1, and CTLA4 (cytotoxic T lymphocyte antigen 4), cancer is not eliminated from the body, but actually survives [55,81,82]. Both driver and passenger mutations can lead to the production of tumor-specific neoantigens by cells, which can be recognized by T lymphocytes tasked with detecting foreign invaders in the body [83,84,85]. CD8+ tumor-infiltrating lymphocytes that are reactive to clonal neoantigens were identified in non-small cell lung cancer and expressed high levels of PD-1 [51]. Sensitivity to PD-1 and CTLA-4 blockade in patients with advanced NSCLC (non-small cell lung cancer) and melanoma was enhanced in tumors enriched for clonal neoantigens [51]. Recent studies have provided insights into the effect on ITH on the immune response, and have shown that the mutational load in combination with ITH is a better predictor of response to checkpoint blockers than the neoantigen burden alone [21,42,50]. Although ITH presents a challenge for conventional and targeted therapies, increased mutational diversity may provide a beneficial opportunity for immunotherapies by generating potential neoantigens that can be recognized by T cells [86] (Figure 4). Highly heterogeneous tumors are possibly driven by neutral evolution, resulting in many passenger mutations, but potentially generate neoantigens that are capable of eliciting an immune response [21,42].

Thus, a promising therapeutic strategy taking ITH into consideration is a combination of targeted therapy directed against driver mutations and immunotherapy (Figure 4). In fact, the authors have demonstrated that a combination of molecular targeted therapy for driver mutations and anti-PD-1 antibody therapy could be an effective therapeutic strategy for sarcomatoid cancers of the lung [14].

### 6.2. Adaptive Therapy

**Figure 5:**
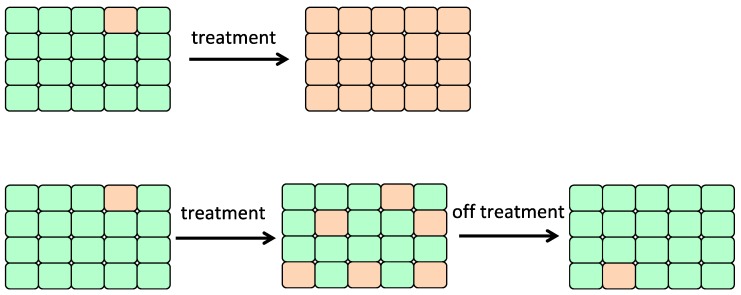
Adaptive therapy. In a maintenance phase of adaptive therapy, drug doses are reduced or chemotherapy is terminated, allowing drug sensitive cells to outcompete resistant subclones. Green cell: drug sensitive cell, orange cell: drug resistant cell.

Another therapeutic strategy based on cancer genome evolution is adaptive therapy. In this therapy, when tumors begin shrinking because of chemotherapy, drug doses are reduced [87,88]. Cells sensitive to chemotherapy are allowed to remain at a certain level and compete with drug-resistant cells to prevent tumor growth (Figure 5) [87,88].

A number of successful systemic therapies are available for the treatment of disseminated cancers. However, the tumor response is often transient, and therapy frequently fails due to the emergence of resistant populations (Figure 5), which mainly reflects the temporal and spatial heterogeneity of the tumor microenvironment. Although cancers have highly dynamic systems, cancer therapy is conventionally administered according to a fixed, linear protocol. While the goals of therapy in standard clinical practice are to maximally reduce tumor burden, the focus of adaptive therapy is to extend the time to progression by stabilizing the tumor size [88,89]. Adaptive therapy entails varying the drug dosing and schedules in two distinct phases: an induction phase, in which tumor progression is slowed, and a maintenance phase, which often requires progressively lower dosing or even omitted schedules in order to achieve better progression-free survival times compared to fixed dosing (Figure 5) [87,88]. It has also been pointed out that clones in recurring tumors may have existed as minor clones since the time of initial treatment [90,91,92]. It is proposed that the timing of administering drugs and their doses should be considered in relation to the compositional balance between sensitive and resistant cancer cells (Figure 5). Gatenby et al. have reported approaches in animal models that take advantage of the fitness cost of resistant subclones by the maintenance of a stable population of sensitive subclones in order to restrict the growth of resistant cells [89].

### 6.3. Reshaping Tumor Evolution

**Figure 6:**
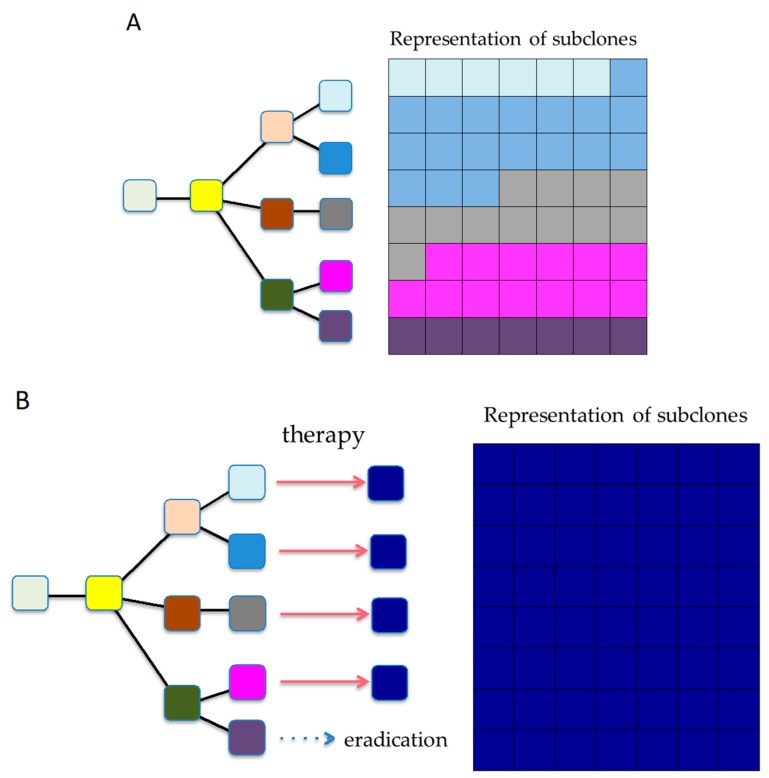
Strategies for countering ITH. (**A**) ITH at time of diagnosis. ITH exists, allowing for tumor evolution. (**B**) Shaping evolution. Rationally, sequencing therapies might allow us to actively shape tumor evolution.

A more difficult but intriguing mode of attack would be to actively shape cancer genome evolution by reducing karyotypic heterogeneity to a defined predictable state through initial drug exposure, which can then be targeted by a secondary drug (Figure 6). Here, we adduce an example of ALK (anaplastic lymphoma kinase)-rearranged NSCLC, a patient who progressed on crizotinib due to a founder ALK C1156Y mutation, but responded to the third-generation ALK TKI lorlatinib. At the time of relapse, this patient was found to have developed an additional ALK mutation (L1198F) that changed the residue that enhanced the selectivity of lorlatinib (but not crizotinib) for ALK over other kinases. This mutation resulted in lorlatinib resistance, yet paradoxically restored sensitivity to crizotinib [93]. Niederst et al. reported similar acquired resistance mechanisms in vitro, and demonstrated that the emergence of C797S in EGFR (epidermal growth factor receptor), a novel mechanism of acquired resistance to third generation tyrosine kinase inhibitor, affects the efficacy of subsequent treatments [94].

While these were fortuitous discoveries, they inspire us to think that rational drug design and selection might allow us to sculpt tumor evolution. These studies illustrate how evolutionary constraints and collateral sensitivity can be exploited for patient benefit. Moreover, in this age, there may be an urgent need to further develop liquid biopsy techniques with high sensitivity that allow the observation and detection of real-time changes in clonal composition [72].

## 7. Conclusions and Future Directions

As described above, it has been elucidated that the evolutionary processes themselves, which are the driving force for cancer genomic heterogeneity, vary among and within cases. The determinants in these evolutionary processes are associated with various elements such as the degree of DNA instability and host immunity, in addition to tumor conditions, such as cellular characteristics, organ environment, and abnormalities in driver genes. If the selection pressure determined by these elements is strong, only a limited number of clones can survive. In contrast, if selection pressure is weak, diverse clones will coexist, and heterogeneity will increase. The condition that produces the highest heterogeneity is the absence of selection pressure or the state of neutral evolution. This condition is maintained in some types of solid tumors, such as lung cancer [15]. However, even in a saturated condition that does not require driver mutations with positive effects on proliferation, there is presumably a certain level of negative selection pressure, such as surveillance by the host immune system or the allowable limiting point for excessive genomic abnormalities. Thus, the neutral evolution of cancer may be in dynamic equilibrium rather than stable equilibrium. In the future, new therapeutic strategies to overcome ITH are expected to be developed by understanding the diversity in cancer from these new perspectives.

In this review, we propose three evolutionary therapeutic approaches: (i) the combination of molecular targeted therapy and immunotherapy, (ii) adaptive therapy, and (iii) active shaping of the cancer genome evolution. Further progress in computation and technology, in tandem with prospective longitudinal studies exploring both the cancer genome and the immune microenvironment, are needed to better understand the evolutionary life history of tumors, with a special emphasis on the next step in their evolutionary course [95]. Such studies are expected to gain insight into both the processes that generate diversity and how constraints on these processes might be exploited, with a resulting proactive management of cancers by controlling the adaptive immune response.
